# Solvent-free automated thermal desorption-gas chromatography/mass spectrometry for direct screening of hazardous compounds in consumer textiles

**DOI:** 10.1007/s00216-023-04780-x

**Published:** 2023-06-20

**Authors:** Josefine Carlsson, Tim Åström, Conny Östman, Ulrika Nilsson

**Affiliations:** Department of Materials and Environmental Chemistry, Stockholm University, SE-106 91 Stockholm, Sweden

**Keywords:** ATD-GC/MS, Textiles, Arylamines, Quinoline, Halogenated nitroaromatic compounds, Skin sensitizer

## Abstract

The global production of textiles utilizes numerous large-volume chemicals that may remain to some extent in the finished garments. Arylamines, quinolines, and halogenated nitrobenzene compounds are possible mutagens, carcinogens and/or skin sensitizers. For prevention, control of clothing and other textiles must be improved, especially those imported from countries without regulations of textile chemicals. An automated analytical methodology with on-line extraction, separation, and detection would largely simplify screening surveys of hazardous chemicals in textiles. Automated thermal desorption-gas chromatography/mass spectrometry (ATD-GC/MS) was developed and evaluated as a solvent-free, direct chemical analysis for screening of textiles. It requires a minimum of sample handling with a total run time of 38 min including sample desorption, chromatographic separation, and mass spectrometric detection. For most of the studied compounds, method quantification limit (MQL) was below 5 µg/g for 5 mg of textile sample, which is sufficiently low for screening and control of quinoline and arylamines regulated by EU. Several chemicals were detected and quantified when the ATD-GC/MS method was applied in a limited pilot screening of synthetic fiber garments. A number of arylamines were detected, where some of the halogenated dinitroanilines were found in concentrations up to 300 µg/g. This is ten times higher than the concentration limit for similar arylamines listed by the EU REACH regulation. Other chemicals detected in the investigated textiles were several quinolines, benzothiazole, naphthalene, and 3,5-dinitrobromobenzene. Based on the present results, we suggest ATD-GC/MS as a screening method for the control of harmful chemicals in clothing garments and other textiles.

**Graphical abstract:**

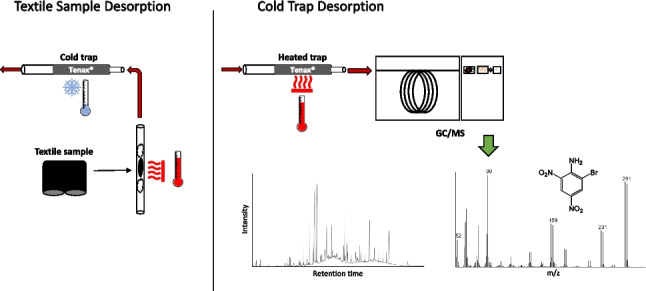

**Supplementary Information:**

The online version contains supplementary material available at 10.1007/s00216-023-04780-x.

## Introduction

The world-wide production of textiles utilizes large volumes of hazardous chemicals, of which several have been shown to remain in finished garments [1]. The human health concern regarding textiles has so far been mostly associated with skin irritation or allergic contact dermatitis. For the latter condition, disperse azo dyes have been considered to be the main culprits [2, 3], but there are also other suspected health effects, such as mutagenicity, reproductive effects, and cancer, which are associated with chemicals present in textiles. Disperse azo dyes comprise some of the most used dyes for synthetic fibers such as polyester, cellulose acetate, and nylon fibers [4]. It is well-known that disperse azo dyes can be metabolized in human skin and/or be degraded by skin bacteria to arylamines [5–8], of which several have shown possible mutagenic and/or carcinogenic effects, as well as skin sensitization potencies [9–11]. We have recently shown that arylamines are frequently abundant in synthetic clothing garments and may occur in levels of several mg/g [12]. These compounds can occur as free compounds [12, 13], not just as reduction products from azo dyes [9, 14–16]. These free arylamines are non-covalently attached to the textile fibers and may thus migrate from skin-close garments to the skin. According to our previous screening survey of synthetic clothing on the Swedish market, the levels of arylamines are generally higher than the concentrations of azo dyes [12], possibly indicating a higher health risk. The high abundance of arylamines is probably mainly due to the common use of impure dyes, including dye precursors, in the manufacturing process. Quinoline and methyl derivatives of quinolines are other frequently detected chemicals in clothes [12, 13, 17]. Quinoline and twenty-two arylamines are regulated by REACH mainly due to their human carcinogenic properties [18]. However, the majority of substances in these two compound classes is still not regulated, but is possibly constituting similar health risks. Halogenated nitroaromatic compounds, of which 2,4-dinitrochlorobenzene (2,4-DNCB) is a well-known and extreme skin sensitizer, are other hazardous non-regulated dye impurities that have been detected in clothing garments [12].

The molecular weights and lipophilic properties (the latter expressed as logP, the logarithm of the partition coefficient between octanol and water) of many quinolines, arylamines, and halogenated nitroaromatic compounds are within ranges that enable skin absorption. Compounds with a molecular weight below 500 Da and log P between 1 and 4 are considered to be most easily absorbed through skin, with a maximum around a log P value of 2. In Table 1, the log P values are listed for the textile chemicals included in the present study. Everyday exposure to skin-close clothes containing high levels of these hazardous compounds may constitute an important human health risk. For prevention, there is a need for more knowledge and awareness regarding chemicals of health concern in new and recycled consumer textiles. Control of these semi-volatile chemical compound classes requires analytical methods for separation and detection, such as gas chromatography/mass spectrometry (GC/MS). There are already analytical methods available for arylamines being released from chemical or bacterial reduction of disperse azo dyes. These methods involve either GC/MS for separation and detection, as described in ISO 14362-1:2017, or liquid chromatography/MS (LC/MS) [15, 16]. Prior to the analyses, the textiles have to be extracted by methods such as ultrasonication-assisted solvent extraction [13, 17]. Direct analysis of textiles without prior solvent extraction is uncommon, but ambient MS, such as “Direct Analysis in Real Time” MS (DART-MS), has been demonstrated for non-quantitative screening of textiles [19].

**Table 1:** Reference compounds used in this study. Retention times (RT) are for ATD-GC/MS. MDLs are for textile samples of 5 mg

	CAS No	logP^a^	RT	Selected ion	LOD/LOQ	MDL/MQL
			[min]	[m/z]	[ng]	[µg/g]
Internal standards (IS)						
Quinoline-d_7_	34071–94-8		7.53	136		
Indanone	83–33-0		8.13	132		
3-Nitroaniline-d_4_	115044–52-5		10.73	96		
Diethyl phthalate-d_4_	93952–12-6		11.96	153		
4-Nitroaniline-^15^N_2_	119516–81-3		12.36	140		
Benzophenone-d_10_	22583–75-1		12.44	110		
Bis(2-ethylhexyl) phthalate-d_4_	93951–87-2		20.77	153		
Analytes						
Naphthalene	91–20-3	3.3	6.84	128	0.39/1.3	0.08/0.26
4-Chloroaniline	106–47-8	1.9	7.08	127	0.81/2.7	0.16/0.54
2-Bromoaniline	615–36-1	2.1	7.22	171	0.72/2.4	0.14/0.48
Benzothiazole	95–16-9	2.0	7.40	135	0.08/0.27	0.02/0.05
Quinoline	91–22-5	2.0	7.57	129	0.36/1.2	0.07/0.24
Isoquinoline	119–65-3	2.1	7.89	129	0.31/1.0	0.06/0.2
3-Bromoaniline	591–19-5	2.1	8.30	171	1.8/5.9	0.35/1.17
4-Bromoaniline	106–40-1	2.3	8.36	171	1.8/5.9	0.35/1.2
2-Methylquinoline	91–63-4	2.6	8.51	143	0.33/1.1	0.07/0.22
2-Amino-4-cresol	95–84-1	1.2	8.62	123	57/190	11/38
8-Methylquinoline	611–32-5	2.6	8.64	143	0.28/0.94	0.06/0.19
6-Methylquinoline	91–62-3	2.6	9.15	143	0.5/1.7	0.1/0.33
3-Methylquinoline	612–58-8	2.5	9.21	143	0.42/1.4	0.08/0.28
4-Methylquinoline	491–35-0	2.6	9.41	143	0.54/1.8	0.11/0.36
2-Nitroaniline	88–74-4	1.9	9.79	138	0.59/2.0	0.12/0.4
2,6-Dimethylquinoline	892874–63-4	3.0	9.97	157	0.4/1.3	0.08/0.27
3,4-Dichloroaniline	95–76-1	2.7	10.07	161	0.96/3.2	0.19/0.64
2,4-Dimethylquinoline	1198–37-4	3.0	10.22	157	0.48/1.6	0.1/0.32
3-Nitroaniline	99–09-2	1.4	10.75	138	5.2/17	1.0/3.5
2,5-Dinitrochlorobenzene	619–16-9	2.1	11.39	202	1.6/5.4	0.33/1.09
2,4-Dinitrochlorobenzene	97–00-7	2.3	11.65	202	1.7/5.6	0.34/1.13
2,6-Dichloro-benzenediamine	609–20-1	2.4	11.99	176	6.6/21.8	1.3/4.4
5-Chloro-2-nitroaniline	1635–61-6	2.7	12.05	172	5.1/17	1.0/3.4
4-Chloro-2-nitroaniline	89–63-4	2.7	12.11	172	3.3/11	0.66/2.2
4-Nitroaniline	100–01-6	1.4	12.25	138	6.0/20	1.2/4.0
3,5-Dinitrobromobenzene	18242–39-2	2.0	12.29	245	1.7/5.7	0.34/1.1
Diphenylamine	122–39-4	3.5	12.36	169	0.45/1.5	0.09/0.3
2-Chloro-4-nitroaniline	121–87-9	2.1	13.20	172	2.5/8.3	0.5/1.7
2,6-Dichloro-4-nitroaniline	99–30-9	2.9	13.44	206	0.66/2.2	0.13/0.44
2-Chloro-4,6-dinitroaniline	3531–19-9	2.1	15.38	217	5.0/17	1.01/3.4
2,6-Dibromo-4-nitroaniline	827–94-1	3.2	15.52	266	1.0/3.4	0.21/0.69
2,4-Dinitroaniline	97–02-9	1.0	16.14	183	28/94	5.6/19
2-Bromo-4,6-dinitroaniline	1817–73-8	2.1	16.37	261	9.4/31	1.9/6.2

^a^Data from PubChem

Avoidance or reduction of solvents in the sample preparation step of the textile analysis is desirable for sustainable and environmental reasons. Automated thermal desorption (ATD) coupled on-line with GC/MS is a solvent-free methodology for direct analysis of volatile [20, 21] and semi-volatile substances ranging up to C_26_ and is common for analysis of adsorbents used for air sampling [22–24], but has also been used for other applications [25–29]. The material to be analyzed is loaded into a tube for thermal desorption. An inert gas flow is applied through the heated tube, and compounds adsorbed on the sample are desorbed by a dynamic extraction process. The desorbed chemical vapor is in the next stage focused in a cold-trap containing a suitable adsorbent, such as Tenax® or Carbograph™. After completed extraction, the material trapped in the cold trap is desorbed and transferred to the GC by fast heating and reversal of the inert gas flow.

In the present study, we have developed a solvent-free, easy-to-handle analytical method based on ATD-GC/MS for direct screening of health hazardous chemical classes in textiles. The main focus has been to be able to identify and quantify compound classes previously identified in clothing garments, such as quinolines, arylamines, and halogenated nitroaromatic compounds. A number of common types of garments from the Swedish market were investigated with ATD-GC/MS, and the performance of this novel textile screening method was compared with off-line analysis including solvent-extraction, solid-phase extraction (SPE) clean-up, and GC/MS.

## Materials and methods

### Chemicals and materials

Acetonitrile, methanol, acetone (all of HPLC grade), and dichloromethane (puriss) were obtained from Honeywell Research Chemicals Ltd (Morris Plains, NJ, USA). Toluene (pesticide grade) was purchased from Rathburn Chemicals Ltd (Walkerburn, Scotland). Deactivated (silanized) glass wool of pesticide grade was obtained from Supelco (Bellefonte, PA). The compounds investigated with the ATD-GC/MS screening method are listed in Table 1. Internal standards and reference compounds were supplied from Sigma-Aldrich, except for quinoline-d_7_ (Cambridge Isotopes laboratories Inc., Tewksbury, MA), quinoline (Merck, Darmstedt, Germany), 3,5-dinitrobromobenzene (Apollo Scientific, Stockport, UK), and 2,5-dinitrochlorobenzene (Toronto Research Chemicals, Toronto, Canada). All analytes in Table 1 were of  > 90% purity, except for 3,5-dinitrobromobenzene and 2,5-dinitrochlorobenzene, which had no purity specified. Four disperse dyes were purchased from Sigma-Aldrich. Disperse Orange 1 and 25 had a purity  > 90%, while Disperse Brown 22 and Disperse Blue 79 had no purity specified. SPE cartridges containing 500 mg of porous graphitized carbon (Carbograph 5, mesh size 120/400, surface area 240 m^2^/g) were purchased from LARA Srl (Formello, Italy). The ATD cold trap packed with Tenax® was purchased from Perkin Elmer Inc. (Waltham, MA, USA).

### Standard solutions

Stock solutions of reference compounds were prepared in acetonitrile, bubbled with argon and stored at  − 20 °C. Working solutions of the analytes, 4 ng/µL of each compound in acetonitrile, were prepared fresh weekly and stored at  − 20 °C. No degradation of the reference compounds was observed during storage. An internal standard solution (IS) used for spiking of samples contained 4 ng/µL each of quinoline-d_7_, 3-nitroaniline-d_4_, benzophenone-d_10_, 8 ng/µL of 4-nitroaniline-^15^N_2_, and 10 ng/µL each of diethyl phtalate-d_4_ and bis(2-ethylhexyl) phthalate-d_4_. For quantification, quinoline-d_7_ was used for quinolines and benzothiazole, and 3-nitroaniline-d_4_ and 4-nitroaniline-^15^N_2_ were used for both arylamines and halogenated dinitrobenzenes. As volumetric standard, a solution of indanone was used at a concentration of 4 ng/µL.

### Samples

Four dark-colored garments were investigated in this study, a black sports jacket from China made of 100% polyester, a blue sports T-shirt from Indonesia made from 91% polyester/9% elastane, a black pair of sports shorts from Vietnam made of 88% polyester/12% elastane, and a black pair of sports trousers from China made of 100% polyester. The garments were bought from common low- to mid-price stores in Stockholm, Sweden. Each garment was analyzed in triplicate, and one-point calibration within the linear range was used for quantification.

Two textiles, “Textile 1” made of 100% polyester and “Textile 2” made of 91% polyester/9% elastane, were used for evaluation of the method.

### Direct analysis with ATD-GC/MS

A part of each garment was cut into as small pieces as possible with the use of scissors. An amount of 5–8 mg of the textile was placed between two plugs of glass wool in a thermal desorption glass tube (Perkin Elmer). The glass wool, preconditioned for 15 min at 300 °C, was used to hold the sample in place, as well as prevent from contamination of the ATD with solid material being released from the sample during the desorption process. A 10-µL volume of the IS solution was spiked onto the textile sample inside the desorption tube. The textile sample was then left to dry in the tube for approx. 30 min prior desorption in the ATD.

#### The ATD-GC/MS setup

**Fig. 1:**
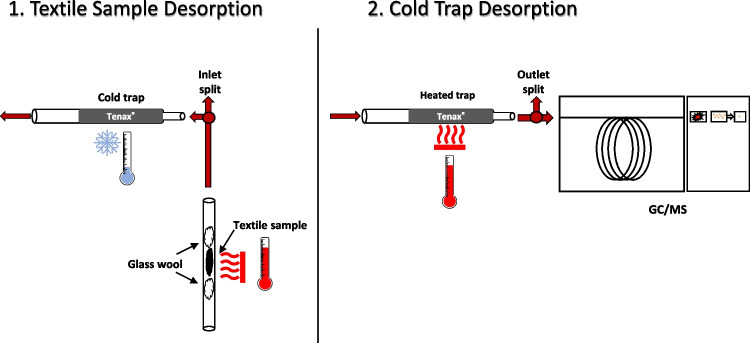
A schematic of the ATD-GC/MS system. (1) Thermal desorption of the sample and cryo-focusing of desorbed chemicals. (2) Heating of the cold-trap and transfer of the chemicals to the GC/MS

A schematic of the ATD-GC/MS method is shown in Fig. 1. The instrument consisted of a TurboMatrix 350 automated thermal desorber (ATD) connected to a Clarus® 680 gas chromatograph/Clarus® SQ 8 T mass spectrometer (Perkin Elmer). The system had two optional split points, before and after the trap, allowing the use of larger sample sizes without risk of overloading the GC system. The run parameters of the ATD were as followed: purge time 1 min; 10 min sample desorption at 175 °C with a desorption flow of nitrogen at 50 mL/min, and an inlet split of 49 mL/min. During sample desorption, the trap temperature was held at − 10 °C. The captured compounds were then desorbed from the trap with a temperature ramp of 40 °C/s up to 230 °C, which was held for 5 min with valve and transfer line temperatures at 250 °C. The outlet split between the trap and the GC inlet was set to 10 mL/min and the GC column helium flow to 1.5 mL/min, resulting in a total trap desorption flow of 11.5 mL/min. The GC column was a 40-m DB-5MS (Agilent Technologies, Palo Alto, CA, USA) with an integrated 10-m guard column (i.d. = 0.25 mm, film thickness = 0.25 µm). The GC oven program started at 50 °C, kept for 0.5 min, followed by a temperature ramp of 10 °C/min up to 325 °C, held for 10 min, resulting in a total run time of 38 min. The first analysis of the ATD-GC/MS sequence took 49 min including 11 min of sample desorption. Since desorption of the subsequent samples was performed while the GC was running, the total run time for each of those was only 38 min. The ion source was held at 190 °C and the MS transfer line temperature was set to 250 °C, and electron ionization (EI) was applied using an electron energy of 70 eV. For identification, the MS was operated in full-scan mode between m/z 50 to 500 with a scan time of 230 ms and an interscan delay of 100 ms. For quantification, the areas of selected extracted ions (m/z) shown in Table 1 were used. The filament was off during the first 6.7 min.

### Preparation of textile solvent extracts

Triplicate samples of 1 g each of Textile 1 and Textile 2 were cut into small pieces of approx. 3 × 3 mm. Each sample was spiked with 100 µL of the IS solution. The IS solution was left to dry for 30 min prior to extraction. The solvent extraction was performed by 15 min of ultrasonication in 10 mL of dichloromethane at 40 °C (Sonorex Digital 10P, BANDELIN Electronic, Berlin, Germany). The ultrasonication was repeated twice with fresh solvent. The extracts were pooled and the solvent evaporated to approx. 1 mL at 40 °C using a gentle stream of N_2_. To remove non-volatile dyes, the extracts were run through a Carbograph 5 SPE cartridge [13, 30]. Conditioning of the cartridge was performed with 10 mL of methanol followed by 5 mL of dichloromethane/toluene (8:2 v/v). The extracts (~ 1 mL) were loaded onto the cartridge and slowly eluted by gravitational force using 15 mL of dichloromethane/toluene (8:2). The eluate was reduced to approx. 1 mL at 40 °C using a gentle stream of N_2_ and then filtered through cellulose syringe filter (0.2 µm RC filter, Phenomenex, Torrance, CA, USA). Prior to analysis, a volume of 100 µL of volumetric standard solution (1-indanone) was spiked into the vials. The recoveries were evaluated by spiking standards to a blank sample made of 100% white polyester (see Supplementary Information, Figure SI-1).

### GC/MS of solvent extracts

The GC/MS system consisted of a 6890N gas chromatograph, a 7683 autosampler, a programmable temperature vaporizer (PTV) injector, and a 5975C mass spectrometer (Agilent Technologies, Palo Alto, CA, USA). The PTV program was as follows: 90 °C for 0.5 min followed by a ramp of 700 °C/min up to 300 °C, which was held for 8 min. The GC oven program was as follows: 90 °C for 0.5 min, followed by a ramp of 10 °C/min up to 325 °C, which was kept for 15 min, resulting in a total run time of 43 min. A J&W CP-Sil 8 CB base-deactivated column was used (stationary phase 5% phenyl/95% polydimethylsiloxane, length 30 m, i.d. 0.25 mm, film thickness 0.25 µm, Agilent Technologies). The MS was operated in EI mode with an electron energy of 70 eV. The MS source temperature and transfer line were both set to 300 °C and the quadrupole analyzer to 200 °C. For identification, the acquisition was performed in full-scan mode between m/z 50 and 500 in 267 ms. The quantification was performed using the area of extracted m/z, as for quantification with ATD-GC/MS (Table 1). The textile solvent extracts (“Preparation of textile solvent extracts” section) were analyzed by injecting 1 µL into the GC/MS system. The accuracy of this off-line method was 88–99% for quinolines and 90–100% for arylamines. The RSD was 0.8–2.1% for quinolines and 0.2–5.3% for arylamines (*N* = 3).

### Comparison of ATD-GC/MS with off-line GC/MS

Solvent extracts of Textile 1 and 2 (“Preparation of textile solvent extracts” section) were also analyzed with ATD-GC/MS for comparison with the off-line method. A 10-µL aliquot of the solvent extract was loaded onto a pre-conditioned piece of glass wool and inserted into the ATD desorption tube. The analyses were performed in triplicate as described in the “The ATD-GC/MS setup” section*.*

### Evaluation of ATD-GC/MS performance

To evaluate linearity, limits of detection/quantification (LOD/LOQ), method detection/quantification limits (MDL/MQL), and repeatability, standards were spiked directly onto glass wool placed in the ATD desorption tubes and then left to dry for approx. 30 min prior to desorption. Instrumental LOD/LOQ were estimated from triplicate injections of a low amount of analyte (2.5, 10 or 80 ng depending on detectability). The area of the peak was defined as the signal (S) and the standard deviation of the triplicate injections as the noise (N). LOD was defined as 3xS/N and LOQ as 10xS/N. The MDL/MQL values were estimated from the instrumental LOD/LOQ based on a 5-mg textile sample. The desorption from spiked blank textile (100% polyester, white) was between 82 and 99% of the desorption from spiked glass wool, except for 2,6-dichloro-4-benzenediamine (49%).

### Data mining

For tentative identification without reference compounds, deconvoluted mass spectra were matched with the MassBank of North America (MoNA) GC/MS spectral library (containing 18902 EI spectra) in the data processing workflow of MS-DIAL [31] with a library match cut-off of 80%. Spectra not matched in MoNA were exported for further matching with the NIST 14 library (ver 2.2, 242466 spectra). In NIST, a reversed-match score  > 850 was used for assigning tentatively identified compounds. Retention indices from injected alkane standards using the same temperature program as for the samples were included in the MS-DIAL workflow to align the detected peaks in the chromatograms and calculate their retention indices as part of the tentative identification.

## Results and discussion

### Selecting ATD parameters

Extraction by thermal desorption is done using elevated temperatures and a flow of inert gas. The standard use of the Perkin-Elmer ATD system is for the desorption and analysis of air sampling adsorbent tubes. To our knowledge, textile screening is a novel application of ATD-GC/MS and may thus require other settings of desorption temperature, time, and gas flows. Type of cold trap adsorbent, focusing temperature, inlet and outlet split ratios, desorption temperature, and time are also pivotal. In the present study, these parameters were investigated with respect to sample extraction efficiency, compound stabilities, carry-over effects, repeatability, linearity, and detection limits. Standard solutions of the target compounds as well as both solvent extracts and solids of two selected textiles were used for this purpose.

#### Adsorbent

Two commercially available cold trap adsorbents were tested, Tenax® and Carbograph™. The latter adsorbent exhibited losses of a number of the selected target compounds, such as methylquinolines, and also gave rise to severe chromatographic peak tailing for some polar analytes. The former adsorbent, on the other hand, was shown to be able to trap and release all tested compounds without any major impact on peak shapes in the GC chromatograms. Thus, Tenax® was the obvious choice for the continuation of the study.

#### Desorption temperature for textile solvent extracts

A temperature range of 175 to 250 °C was evaluated for desorption of a textile solvent extract (*N* = 3) adsorbed on glass wool. As described above, most dyes as well as fibers and particulate material were removed from the textile extract in order to be able to use an “ordinary” off-line GC/MS and compare the results with ATD-GC/MS. In Table 2, the concentrations are shown of all investigated compounds in the two textiles when using the two different systems as well as different temperatures for the ATD. For 2,6-dichloro-4-nitroaniline and 4-nitroaniline in Textile 1, higher levels were observed with increasing temperatures of the ATD and with the GC/MS. This can be explained by dyes still present in the textile solvent extracts. It was found that GCB was unable to remove all dyes, and the SPE resulted in an orange/red-colored extract. The ATD analysis at 250 °C showed a release from the textile of Disperse Orange 30, tentatively identified using the NIST MS library (see Figure SI-2). Further, Disperse Orange 3 was identified, a possible precursor to 4-nitroaniline (see Figure SI-3). By utilizing a previous method for screening with LC/HRMS [12], the presence of these dyes was also suggested in the textile solvent extract (see Table SI-1). The ATD analysis at 175 °C did not exhibit any peaks from these compounds (see Figures SI-4 and SI-5). Increasing levels of 2,6-dichloro-4-nitroaniline and 4-nitroaniline at higher temperatures may thus be due to the thermal degradation of these dyes or any other dyes, e.g., Disperse Brown 22, containing a 2,6-dichloro-4-nitroaniline moiety.

**Table 2:** Solvent extracts from 1 g each of textile 1 (100% polyester) and 2 (91% polyester, 9% elastane), respectively (see “ Samples” and “Preparation of textile solvent extracts” sections), analysed by both ATD-GC/MS at different sample desorption temperatures and off-line GC/MS. Prior to analysis, dyes and particulate material were removed by SPE clean-up. Concentrations in ng/g. *N* = 3

	ATD-GC/MS				GC/MS
	175 °C	200 °C	225 °C	250 °C	
Textile 1					
Benzothiazole	1870 ± 139	1880 ± 178	1920 ± 135	1920 ± 102	1850 ± 130
Quinoline	1130 ± 33	1140 ± 44	1090 ± 19	1080 ± 31	1290 ± 12
Isoquinoline	260 ± 15	280 ± 22	290 ± 5	270 ± 17	380 ± 6
2-Methylquinoline	150 ± 12	160 ± 10	160 ± 9	160 ± 1	170 ± 1
8-Methylquinoline	50 ± 1	60 ± 7	60 ± 10	50 ± 2	50 ± 1
6-Methylquinoline	110 ± 13	120 ± 7	120 ± 18	130 ± 8	140 ± 1
4-Methylquinoline	40 ± 7	40 ± 3	40 ± 5	40 ± 2	40 ± 3
2,6-Dimethylquinoline	40 ± 8	30 ± 6	50 ± 8	50 ± 5	20 ± 0
2,6-Dichloro-benzenediamine	380 ± 13	360 ± 7	820 ± 56	1050 ± 63	1550 ± 131
4-Nitroaniline	1300 ± 24	1380 ± 81	2140 ± 41	3720 ± 183	5040 ± 143
3,5-Dinitrobromobenzene	220 ± 23	190 ± 18	180 ± 7	160 ± 8	320 ± 30
2-Chloro-4-nitroaniline	1620 ± 24	1700 ± 99	1710 ± 33	2010 ± 35	2920 ± 136
2,6-Dichloro-4-nitroaniline	4710 ± 363	6010 ± 621	10410 ± 953	20150 ± 1513	32790 ± 583
2-Chloro-4,6-dinitroaniline	38780 ± 5082	37620 ± 2344	32300 ± 1707	30560 ± 534	54340 ± 681
2,6-Dibromo-4-nitroaniline	10530 ± 1384	9950 ± 735	8210 ± 468	7750 ± 199	11530 ± 256
2,4-Dinitroaniline	960 ± 62	910 ± 68	1050 ± 58	1070 ± 67	1410 ± 130
2-Bromo-4,6-dinitroaniline	3300 ± 441	3110 ± 224	2630 ± 181	2500 ± 113	4150 ± 14
Textile 2					
Naphthalene	130 ± 2	120 ± 17	140 ± 16	160 ± 10	190 ± 8
Benzothiazole	1060 ± 13	1060 ± 57	970 ± 38	1110 ± 44	1080 ± 30
Quinoline	970 ± 55	900 ± 181	880 ± 112	950 ± 88	930 ± 101
Isoquinoline	140 ± 37	110 ± 27	120 ± 18	140 ± 20	180 ± 30
2-Methylquinoline	-	-	-	-	78 ± 14
2,4-Dimethylquinoline	127 ± 7	126 ± 27	116 ± 15	121 ± 3	-
2,6-Dichloro-benzenediamine	4614 ± 137	4498 ± 196	4555 ± 256	5078 ± 318	37377 ± 1082
3,5-Dinitrobromobenzene	880 ± 32	870 ± 92	890 ± 122	1040 ± 60	1230 ± 79
2-Chloro-4-nitroaniline	501 ± 19	524 ± 22	661 ± 52	749 ±	1113 ± 21
2,6-Dichloro-4-nitroaniline	5412 ± 583	9171 ± 1899	18646 ± 2099	33761 ± 5195	111655 ± 13138
2-Bromo-4,6-dinitroaniline	40420 ± 380	42280 ± 1650	41660 ± 2580	41300 ± 3270	37400 ± 1080

##### Desorption temperature for textiles

 The temperature range investigated for desorption when applying direct analysis of textile materials with the ATD-GC/MS system was set to 175–250 °C. The reason for this was that at higher temperatures than 250 °C, it was observed that the polyester textile fibers started to melt, and when increasing the temperature further they formed a compact, discolored plug in the ATD tube.

Table 3 shows the relative levels from repeated desorption of a textile sample. Desorption temperatures of 175 °C and 250 °C were compared. At 175 °C, all compounds were completely extracted using just one desorption run with no carry-over in the 2nd desorption, except for three compounds which were extracted to around 95%. When increasing the temperature to 250 °C, the total amounts of several compounds increased as well as being detected in the 2nd, 3rd, and 4th repeated desorption of the same sample.

**Table 3:** The percentage distribution of the amount of chemical in a black textile made of 100% polyester (textile 1) in repeated direct ATD-GC/MS at 175 and 250 °C. The total amount is normalized to 100%

	Sample desorption at 175 °C				Sample desorption at 250 °C			
Compound	1st	2nd	3rd	4th	1st	2nd	3rd	4th
Benzothiazole	100.0	-	-	-	100.0	-	-	-
Quinoline	100.0	-	-	-	100.0	-	-	-
Isoquinoline	100.0	-	-	-	100.0	-	-	-
2-Methylquinoline	100.0	-	-	-	100.0	-	-	-
8-Methylquinoline	100.0	-	-	-	100.0	-	-	-
6-Methylquinoline	100.0	-	-	-	100.0	-	-	-
3-Methylquinoline	100.0	-	-	-	100.0	-	-	-
4-Methylquinoline	100.0	-	-	-	100.0	-	-	-
2,6-Dimethylquinoline	100.0	-	-	-	100.0	-	-	-
2,6-Dichloro-benzenediamine	93.4	6.6	-	-	59.5	17.7	13.2	9.7
4-Nitroaniline	100.0	-	-	-	42.5	24.9	17.3	15.3
3,5-Dinitrobromobenzene	100.0	-	-	-	39.7	25.9	20.2	14.2
2-Chloro-4-nitroaniline	100.0	-	-	-	34.3	26.2	21.7	17.8
2,6-Dichloro-4-nitroaniline	93.5	3.9	2.6	-	41.4	22.9	18.2	17.5
2-Chloro-4,6-dinitroaniline	92.7	4.6	2.7	-	39.9	22.4	19.6	18.1
2,6-Dibromo-4-nitroaniline	100.0	-	-	-	100.0	-	-	-
2,4-Dinitroaniline	100.0	-	-	-	46.1	25.6	14.2	14.1
2-Bromo-4,6-dinitro-aniline	100.0	-	-	-	44.3	23.0	17.7	14.9

At higher temperatures, there is a risk of thermal degradation of textile azo dyes present in the textile material, which can lead to the formation of arylamines. This is supported by the results shown by Disperse Orange 30 (“Desorption temperature for textile solvent extracts” section). To further verify this hypothesis, 40 µg of Disperse Brown 22, Disperse Orange 25, Disperse Blue 79, and Disperse Orange 1 (which were available as standard reference dyes) were individually subjected to thermal desorption at 175 °C and 250 °C, respectively, in the ATD-GC/MS instrument. Disperse Brown 22 and Disperse Blue 79 could not be detected per se due to their very low volatilities, but three interesting results are demonstrated in Table 4. First, the dyes may contain remains from the synthesis, with 2,6-dichloro-4-nitroaniline in Disperse Brown 22 as an example, which at 175 °C had a content up to 1.2% (w/w) of dye precursor impurities. Second, dyes can degrade and release arylamines at a temperature of 250 °C. This is exemplified by a large increase of 2-bromo-4,6-dinitroaniline emanating from Disperse Blue 79, exhibiting a 27-fold increase when increasing the temperature from 175 to 250 °C. Also Disperse Orange 25 degrades at the higher temperature. At a desorption temperature of 175 °C, 4-nitroaniline could not be detected, while at 250 °C a substantial amount of this dye precursor was formed. Third, the dyes may consist of a mixture of more than one type of dye molecule. The latter is demonstrated by the commercial dye named as Disperse Orange 25 which was shown to generate 2,6-dichloro-4-nitroaniline at 250 °C. Chemically pure Disperse Orange 25 is a molecule without any chlorine atoms in the structure. Thus, this commercial dye does not contain 100% pure Disperse Orange 25. Due to the combined results, the sample desorption temperature for the ATD-GC/MS analysis of textile materials was set to 175 °C.

**Table 4:** Quantified amounts in ng from injected dyes and percentage of impurities and/or degradation products after ATD-GC/MS analysis of some selected disperse azo dyes. *N* = 1

Compound	Disperse Brown 22		Disperse Blue 79		Disperse Orange 1		Disperse Orange 25	
	175 °C	250 °C	175 °C	250 °C	175 °C	250 °C	175 °C	250 °C
2,4-Dinitrochlorobenzene	6.9							
4-Nitroaniline						42		95
3,5-Dinitrobromobenzene			13	62				
Diphenylamine					5.6	4.7		
2-Chloro-4-nitroaniline	17	25						
2,6-Dichloro-4-nitroaniline	437	816	9.5	46		16	2.7	24
2-Bromo-4,6-dinitroaniline			64	1757				
**Sum of impurities**	**461**	**841**	**87**	**1865**	**5.6**	**63**	**2.7**	**120**
**Sum of impurities in %**	**1.2**	**2.1**	**0.22**	**4.7**	**0.01**	**0.2**	**0.01**	**0.3**

##### Desorption time

 The ATD-GC/MS was loaded with 5–8 mg of cut textile pieces. Higher amounts were avoided to prevent contamination of the system. At the selected desorption temperature, 175 °C, sample extractions were made from 10 to 30 min in 5-min intervals. No significant change in extracted chemicals could be detected within this time interval when using Student’s *t*-test at *p* = 0.05 (*N* = 3). Thus, 10 min was selected as the time sufficient for complete sample desorption, and 5 min was selected for subsequent desorption in the cold trap with no statistically significant difference by performing a Student’s *t*-test at *p* = 0.05 between 3-, 5-, and 8-min desorption time.

##### Desorption gas flow and inlet and outlet split

 The desorption and split gas flows were all set to standard values as recommended by the manufacturer, see the “The ATD-GC/MS setup” section*.* There was no improvement in the ATD-GC/MS performance when changing these settings. A decrease in inlet split resulted in unwanted contamination of the ATD system, while a decrease in the outlet split, to try and improve the method detection limits, resulted in deteriorated peak shapes in the GC analysis. An increase in both split flows only resulted in increased detection limits.

##### Detection limits, repeatability, linearity, and carry-over

 Detection limits using the final set of parameters are shown in Table 1. The RSD values for analysis of standards solutions, 40 ng spiked on glass wool, were in the range 8–15% (*N* = 7, except for 2,4-dinitroaniline and 2-amino-4-cresol, both spiked 80 ng, *N* = 3). Only 2-amino-4-cresol showed a higher RSD value, 26%. The responses were linear for all studied compounds within the investigated concentration ranges by at least six levels with *R* values  > 0.959 and random distribution of the residuals. Carry-over was found to be less than 1% for all investigated compounds.

### Comparison of ATD-GC/MS with off-line solvent extraction and GC/MS

ATD-GC/MS analysis of the black-colored Textile 1 at a sample desorption temperature of 175 °C, was compared with the off-line method using solvent extraction, SPE clean-up, and GC/MS as described above. In general, the results agreed well. For three compounds, 2-chloro-4,6-dinitroaniline, 2-bromo-4,6-dinitroaniline, and 2,6-dimethylquinoline, the levels were higher with the ATD-GC/MS analysis (Table 5). The reason could, for instance, be a higher textile extraction efficiency with ATD, but as shown above in the “Desorption temperature for textiles” paragraph, it is not due to thermal degradation of the disperse dyes in the textile.

**Table 5:** Comparison of direct ATD-GC/MS at a desorption temperature of 175 °C with GC/MS of a solvent extract. In both cases, the sample was textile 1. Concentration in ng/g, *N* = 5. Sample amount for ATD-GC/MS was 5 mg and 1 g for off-line GC/MS

Compound	RT (min)	175 °C	GC/MS	Ratio
Benzothiazole	7.4	2240 ± 120	1850 ± 130	1.21
Quinoline	7.6	1570 ± 590	1290 ± 10	1.21
Isoquinoline	7.9	470 ± 140	380 ± 6	1.23
2-Methylquinoline	8.5	230 ± 86	170 ± 1	1.32
8-Methylquinoline	8.7	det	50 ± 1	-
6-Methylquinoline	9.1	det	140 ± 1	-
3-Methylquinoline	9.2	-	50 ± 4	-
4-Methylquinoline	9.4	-	40 ± 3	-
2,6-Dimethylquinoline	10.0	-	20 ± 0.4	-
2,6-Dichloro-benzenediamine	12.0	-	1550 ± 130	-
4-Nitroaniline	12.2	det	5040 ± 143	-
3,5-Dinitrobromobenzene	12.3	det	320 ± 30	-
2,6-Dichloro-4-nitroaniline	13.4	7500 ± 1590	32790 ± 583	0.23
2-Chloro-4,6-dinitroaniline	15.4	85400 ± 8610	54300 ± 680	1.57
2,6-Dibromo-4-nitroaniline	15.5	17200 ± 2970	11500 ± 260	1.49
2,4-Dinitroaniline	15.8	-	1410 ± 130	-
2-Bromo-4,6-dinitroaniline	16.3	det	4150 ± 10	-

To investigate how representative a sample was from a specific garment, we analyzed three different parts of Textile 1 (*N* = 9) by direct analysis with our ATD-GC/MS system (see Figure SI-6). The results show similar levels between three different parts of the textile.

### Screening of synthetic garments with ATD-GC/MS

The total run time with the final ATD-GC/MS method was 38 min. This includes sample desorption, GC separation, and MS detection. Compared to an off-line method including solvent extraction, removal of dyes with SPE, solvent evaporation, and final GC/MS analysis, this gives a direct, faster, and solvent-free analytical method with minimal sample handling and no need for dye removal. The ATD-GC/MS is a robust system that enables a much higher sample throughput than the off-line analysis.

When loading a sample amount of 5 mg textile, MQL was  < 5 µg/g for all investigated compounds, except 2-bromo-4,6-dinitroaniline (6.2 µg/g) and 2,4-dinitroaniline (19 µg/g) (Table 1). For twenty-seven of the thirty-three investigated compounds, MDL was  ≤ 1 µg/g textile**.** These values are well below the REACH limit values of 30 µg/g set by EU for a number of selected individual amines in textiles. The only exception was 2-amino-4-cresol which had an MQL of 38 µg/g. This is due to the comparably high polarity of this compound, making it prone to adsorption on surfaces in the analytical system, which was manifested as strong peak tailing in the GC analysis.

**Fig. 2:**
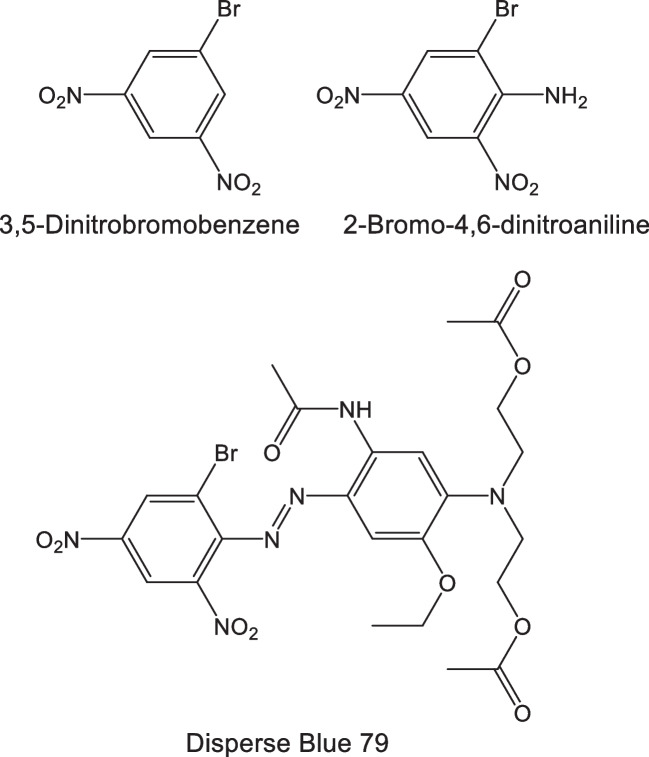
Chemical structures of 3,5-dinitrobromobenzene, 2-bromo-4,6-dinitroaniline and Disperse Blue 79

In a pilot study, four sports garments were analyzed with the developed ATD-GC/MS method, with the results summarized in Table 6. The compounds occurring at the highest levels were 2-chloro-4,6-dinitroaniline and 2-bromo-4,6-dinitroaniline. This is in agreement with a previous study by our group [12], indicating that halogenated anilines are the most abundant arylamines in several dark-colored garments. Both 2-bromo-4,6-dinitroaniline and 3,5-dinitrobromobenzene (Fig. 2) were detected in all four textiles. Due to their structural similarities, these two compounds most likely originate from the same source, e.g., Disperse Blue 79.

**Table 6:** Four sports garments analysed with the direct ATD-GC/MS method. Concentrations are given in µg/g as the mean and standard deviation of three analyses.—= not detected, det = above MDL but below MQL

Compound	Black jacket	Blue T-shirt	Black shorts	Black trousers
Naphthalene	det	det	det	det
Benzothiazole	2.26 ± 0.27	1.06 ± 0.013	5.34 ± 0.60	9.66 ± 0.70
Quinoline	1.46 ± 0.18	0.97 ± 0.06	0.70 ± 0.07	2.18 ± 0.14
Isoquinoline	0.38 ± 0.024	det	0.22 ± 0.05	0.73 ± 0.04
2-Methylquinoline	det	-	det	0.30 ± 0.02
8-Methylquinoline	det	-	det	det
6-Methylquinoline	det	-	det	det
3-Methylquinoline	-	-	det	det
4-Methylquinoline	-	-	det	det
2,6-Dimethylquinoline	-	-	-	det
2,4-Dimethylquinoline	det	det	det	det
2,6-Dichloro-benzenediamine	-	4.61 ± 0.14	-	-
4-Chloro-2-nitroaniline	-	-	-	7.82 ± 1.77
4-Nitroaniline	4.01 ± 0.94	-	21.1 ± 1.94	4.74 ± 0.70
3,5-Dinitrobromobenzene	det	det	1.94 ± 0.22	det
2-Chloro-4-nitroaniline	3.68 ± 0.64	det	det	7.48 ± 1.45
2,6-Dichloro-4-nitroaniline	8.08 ± 1.68	5.41 ± 0.58	0.54 ± 0.07	3.09 ± 0.53
2-Chloro-4,6-dinitroaniline	78.0 ± 13.1	-	114 ± 11.2	116 ± 23.0
2,6-Dibromo-4-nitroaniline	17.1 ± 3.72	-	-	-
2,4-Dinitroaniline	-	-	19.4 ± 1.44	-
2-Bromo-4,6-dinitroaniline	det	40.4 ± 0.38	282 ± 28.1	15.1 ± 3.53

Neither quinoline nor any of the arylamines regulated by REACH could be detected above the allowed limits (50 µg/g for quinoline and 30 µg/g for each of 22 arylamines) (Table 6). However, the two non-regulated compounds, 2-chloro-4,6-dinitroaniline and 2-bromo-4,6-dinitroaniline, both exceeded 30 µg/g and were detected at levels as high as 115 and 282 µg/g, respectively. The latter is almost 10 times the REACH limit and thus constitutes a possible health risk for mutagenicity and/or skin sensitization.

**Fig. 3:**
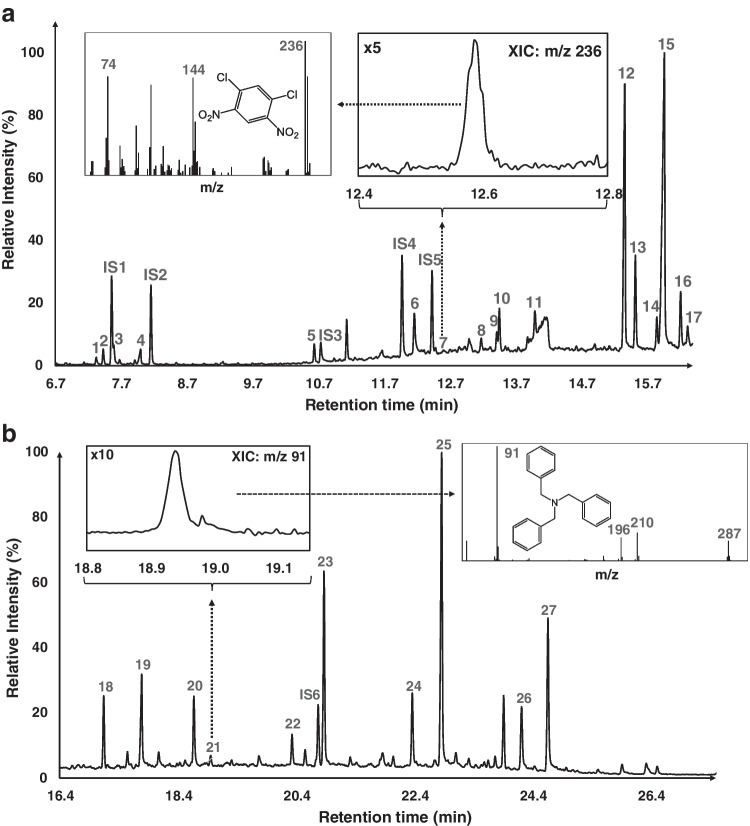
Total ion chromatogram, shown in two different retention time spans, from ATD-GC/MS of a black sports jacket (100% polyester). **a** Time span 6.7–16.4 min with both confirmed and tentatively identified (*) compounds. In retention order: 1 = 2-phenoxyethanol*, 2 = benzothiazole, IS1 = quinoline-d_7_, 3 = quinoline, 4 = nonanoic acid*, IS2 = 1-indanone, 5 = 1-dodecanol*, IS3 = 3-nitroaniline-d_4_, IS4 = diethyl phthalate-d_4_, 6 = 4-nitroaniline, 7 = 1,3-dichloro-4,6-dinitrobenzene*, IS5 = benzophenone-d_10_, 8 = 2-chloro-4-nitroaniline, 9 = ethylene glycol monododecyl ether*, 10 = 2,6-dichloro-4-nitroaniline, 11 = benzyl benzoate*, 12 = 2-chloro-4,6-dinitroaniline, 13 = 2,6-dibromo-4-nitroaniline, 14 = dibutyl phthalate*, 15 = n-hexadecanoic acid*, 16 = diethylene glycol monododecyl ether*, 17 = 2-bromo-4,6-dinitroaniline. The inset shows the peak from extracted ion m/z 236 (1,3-dichloro-4,6-dinitrobenzene*). **b** Time span 16.4–27.4 min with only tentatively identified peaks. In retention order: 18 = N-benzyl-1-phenylethanamine, 19 = octadecanoic acid, 20 = triethylene glycol monododecyl ether, 21 = tribenzylamine, 22 = hexadecanal, IS6 = bis(2-ethylhexyl) phthalate-d_4_, 23 = tetraethylene glycol monododecyl ether, 24 = hexaethylene glycol, 25 = pentaethylene glycol monododecyl ether, 26 = hexaethylene glycol monododecyl ether, 27 = heptaethylene glycol monododecyl ether. The inset shows the peak from extracted ion m/z 91 (tentatively identified as tribenzylamine)

Figure 3a and b show two separate time spans of an ATD-GC/MS chromatogram from a black sports jacket. All analytes identified by reference compounds eluted before 16.4 min. Furthermore, a number of a priori unknown compounds were tentatively identified by data mining using the MS DIAL software for mass spectral deconvolution together with the MoNA and NIST libraries (Fig. 3b). For example, a number of surfactants were detected, e.g., tri-, tetra-, penta-, and hexaethylene glycol monododecyl ether. N-benzyl-1-phenylethanamine, a skin irritant according to ECHA, and tribenzylamine, a skin sensitizer listed in Annex III REACH (EC / List no.: 210-638-3), and a suspected carcinogen, 1,3-dichloro-4,6-dinitrobenzene (EC/List no.: 223-027-1, Annex III, REACH), were tentatively identified in the jacket.

## Conclusions

To our knowledge, this is the first time ATD-GC/MS has been used for direct analysis of textile materials, and this pilot study shows its potential for being used as a high-throughput screening method. Being automated, having minimal sample handling and with low MDL levels compared to the REACH limit values, it constitutes a potential powerful tool for control of harmful chemicals in textiles. The thermal desorption technique is solvent-free, and the method eliminates the use of disposable clean-up devices such as SPE cartridges, which also make the ATD-GC/MS method a more environmentally friendly alternative to common off-line analyses.

Our studies have indicated large variations in both content and levels of quinolines, arylamines, and other health hazardous chemicals in clothing garments. Screening studies are needed to thoroughly map the occurrence of these kinds of compounds in textiles on the market to increase the knowledge regarding the potential exposure. Depending on the occurrence and levels, it is possible that these chemicals constitute an important daily exposure of the general population. As far as we know, there is no or very little control of garments and other textiles that are imported to the European economic area from other countries, explaining why levels reaching mg/g can be detected in several garments sold on the European market.

As indicated in the present study, the developed ATD-GC/MS method is promising for screening, identification and quantification of many classes of semi-volatile chemicals in textiles. Further, using ATD-GC/MS with a non-target approach and data mining has the potential to enable identification of a priori unknown chemicals of toxicological interest.

## Supplementary Information

Below is the link to the electronic supplementary material.

Supplementary file1 (DOCX 608 KB)
