# Supplementary material for: Solvent-free automated thermal desorption-gas chromatography/mass spectrometry for direct screening of hazardous compounds in consumer textiles
Source: Anal Bioanal Chem. 2023 Jun 20;415(19):4675–87. doi: 10.1007/s00216-023-04780-x (PMC10352158; doi:10.1007/s00216-023-04780-x)
Supplement: Supplementary file 1 — Supplementary file1 (DOCX 608 KB) [file 216_2023_4780_MOESM1_ESM.docx]

**Solvent-free automated thermal desorption – gas chromatography/mass spectrometry for direct screening of hazardous compounds in consumer textiles**

Josefine Carlsson*, Tim Åström*, Conny Östman, Ulrika Nilsson

Department of Materials and Environmental Chemistry, Stockholm University, SE-106 91 Stockholm, Sweden

* Shared first authorship

Correspondence: Ulrika Nilsson, Dep of Materials and Environmental Chemistry, Stockholm University, SE-106 91 Stockholm, Sweden.

e-mail: [ulrika.nilsson@mmk.su.se](mailto:ulrika.nilsson@mmk.su.se)

**Supplementary Information**


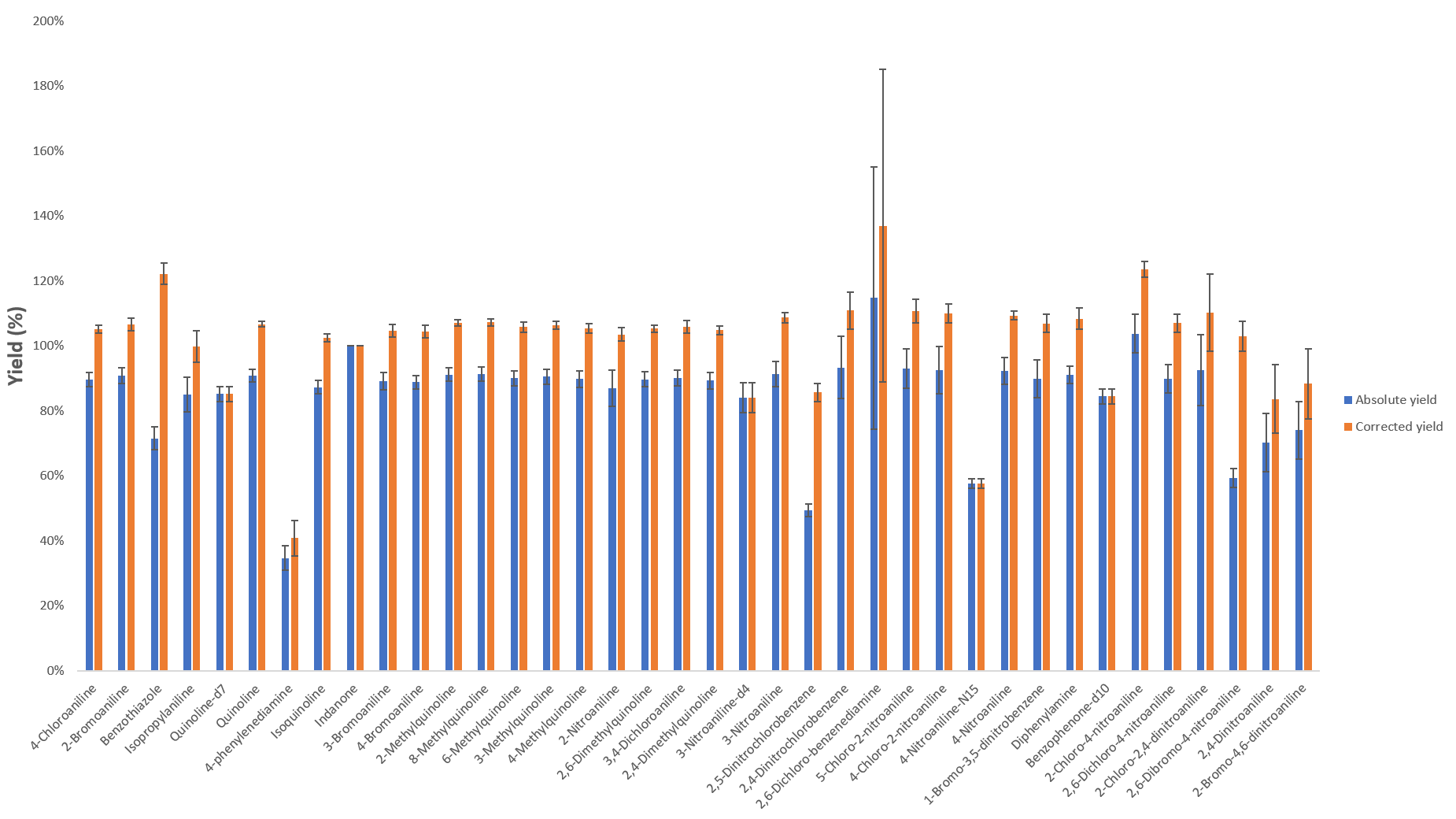


**Figure SI-1:** Matrix-spiked recovery experiments using a blank textile of 100% polyester (N=3) for all investigated compounds using SPE with Carbograph 5 (GCB-5). As a volumetric standard, 1-Indanone was used, to which the absolute recovery has been estimated. Corrected recovery is estimated by considering surrogate internal standards. All compounds are presented in retention order.


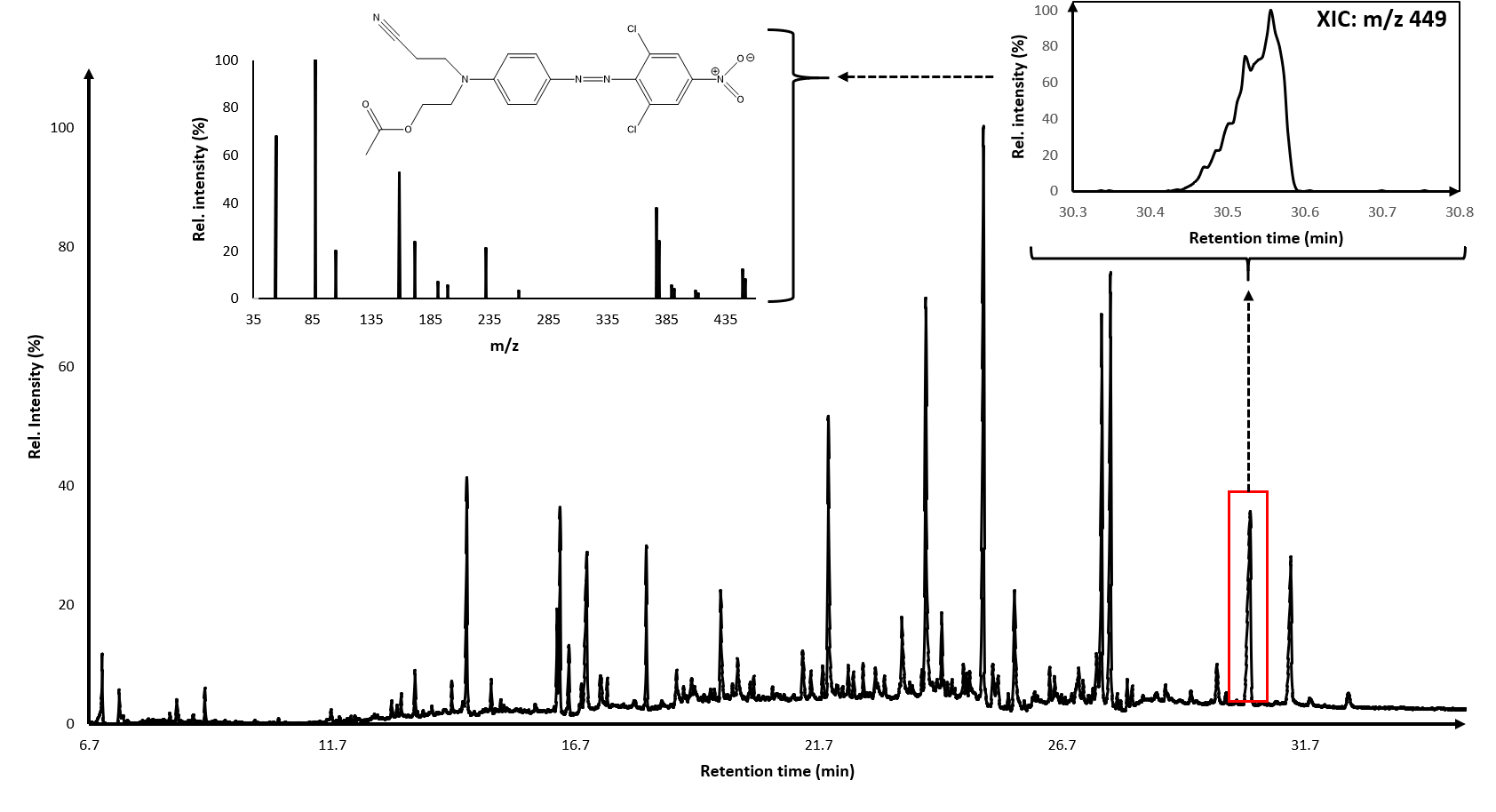


2,6-dichloro-4-nitroaniline

**ATD 250 °C**

**DO30**

**Figure SI-2:** Thermal desorption of Textile 1 at 250°C. The insert shows an extracted ion chromatogram of m/z 449, corresponding to the tentatively identified Disperse Orange 30 according to the NIST 17 MS library.


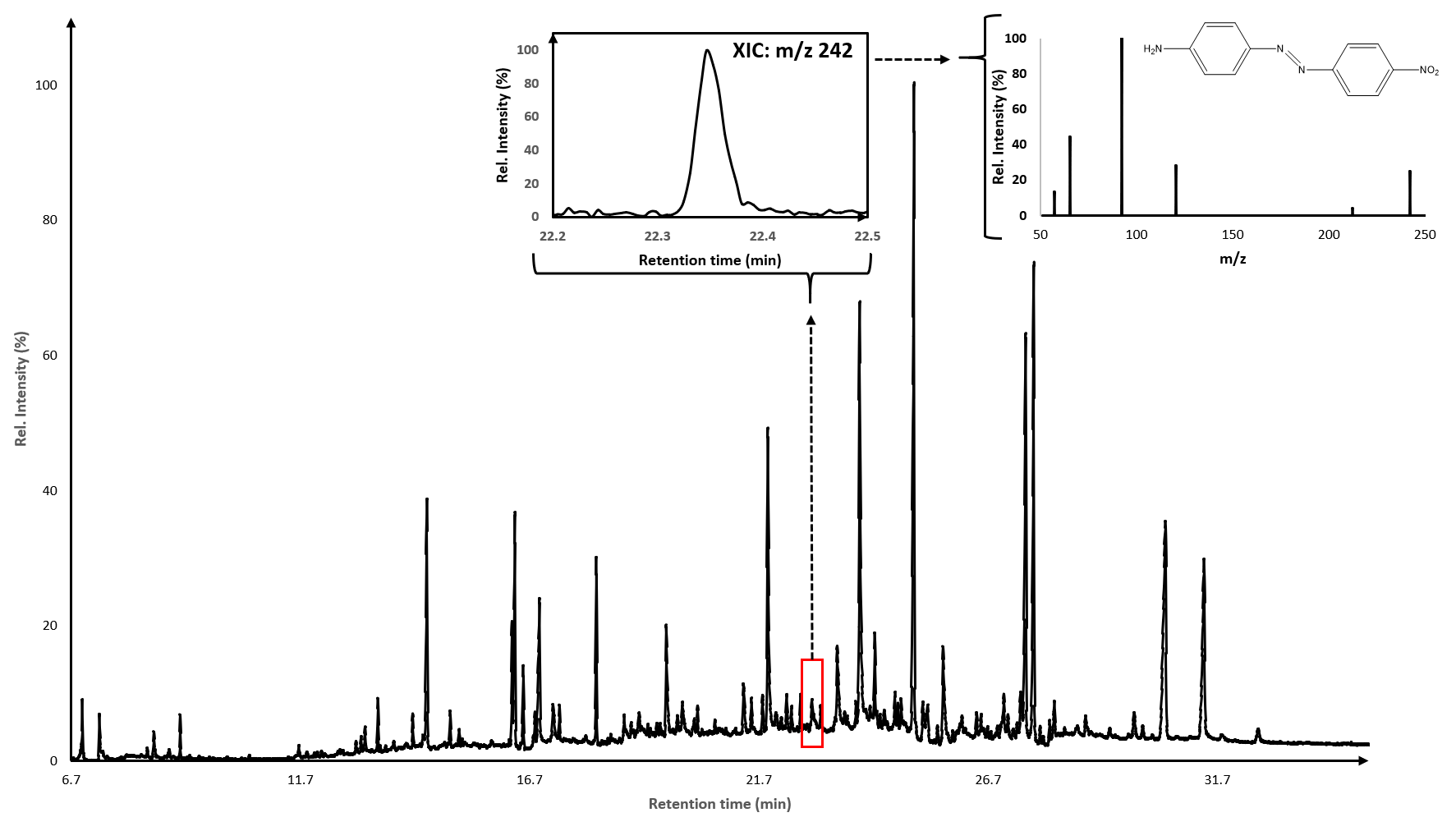


4-nitroaniline

**ATD 250 °C**

**DO3**

**Figure SI-3:** Thermal desorption of Textile 1 at 250°C. The insert shows an extracted ion chromatogram of m/z 242, corresponding to the tentatively identified Disperse Orange 3.


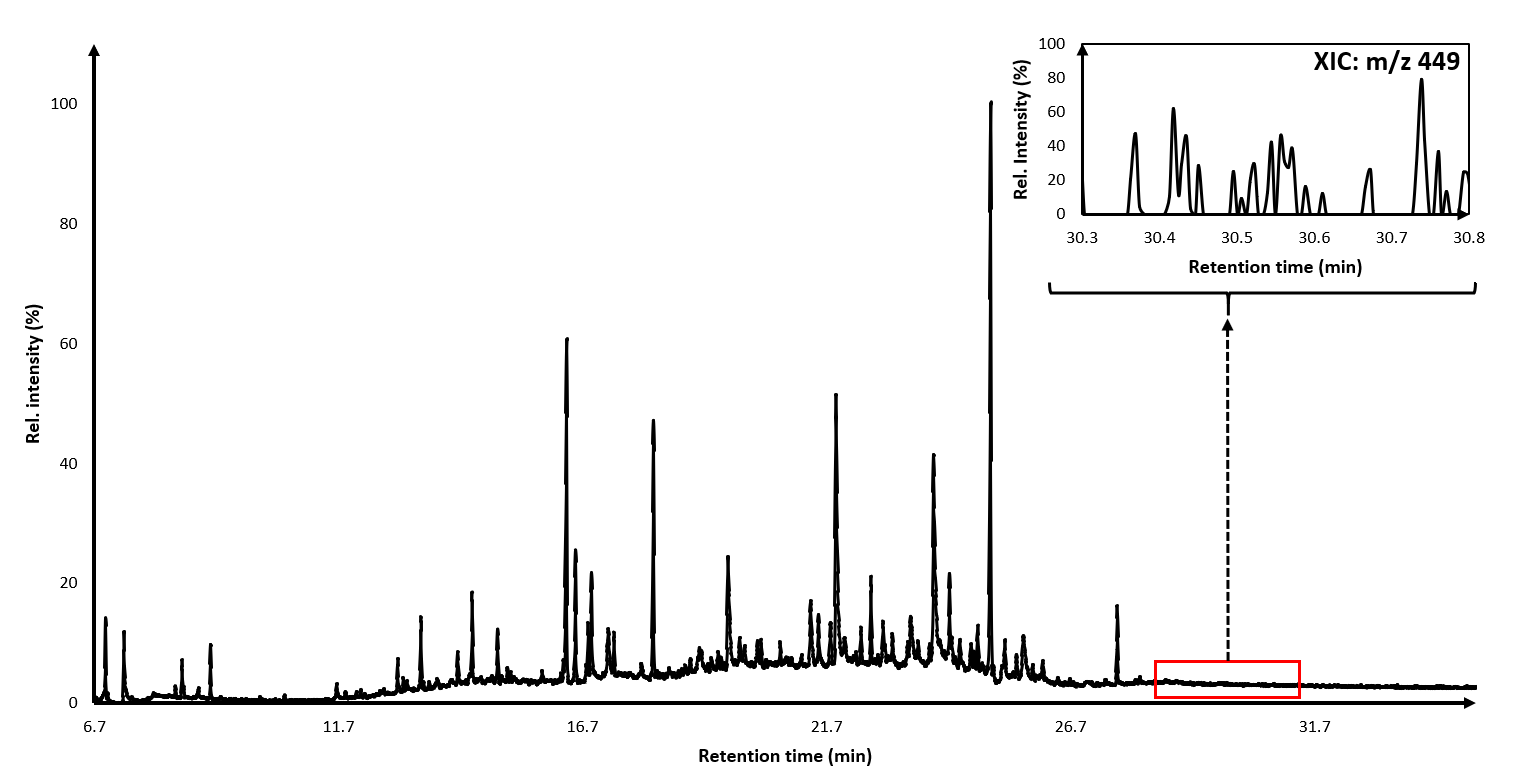


2,6-dichloro-4-nitroaniline

**ATD 175 °C**

**DO30**

**Figure SI-4:** Thermal desorption of Textile 1 at 175°C. The insert shows an extracted ion chromatogram of m/z 449. No peak belonging to Disperse Orange 30 was observed at this desorption temperature.


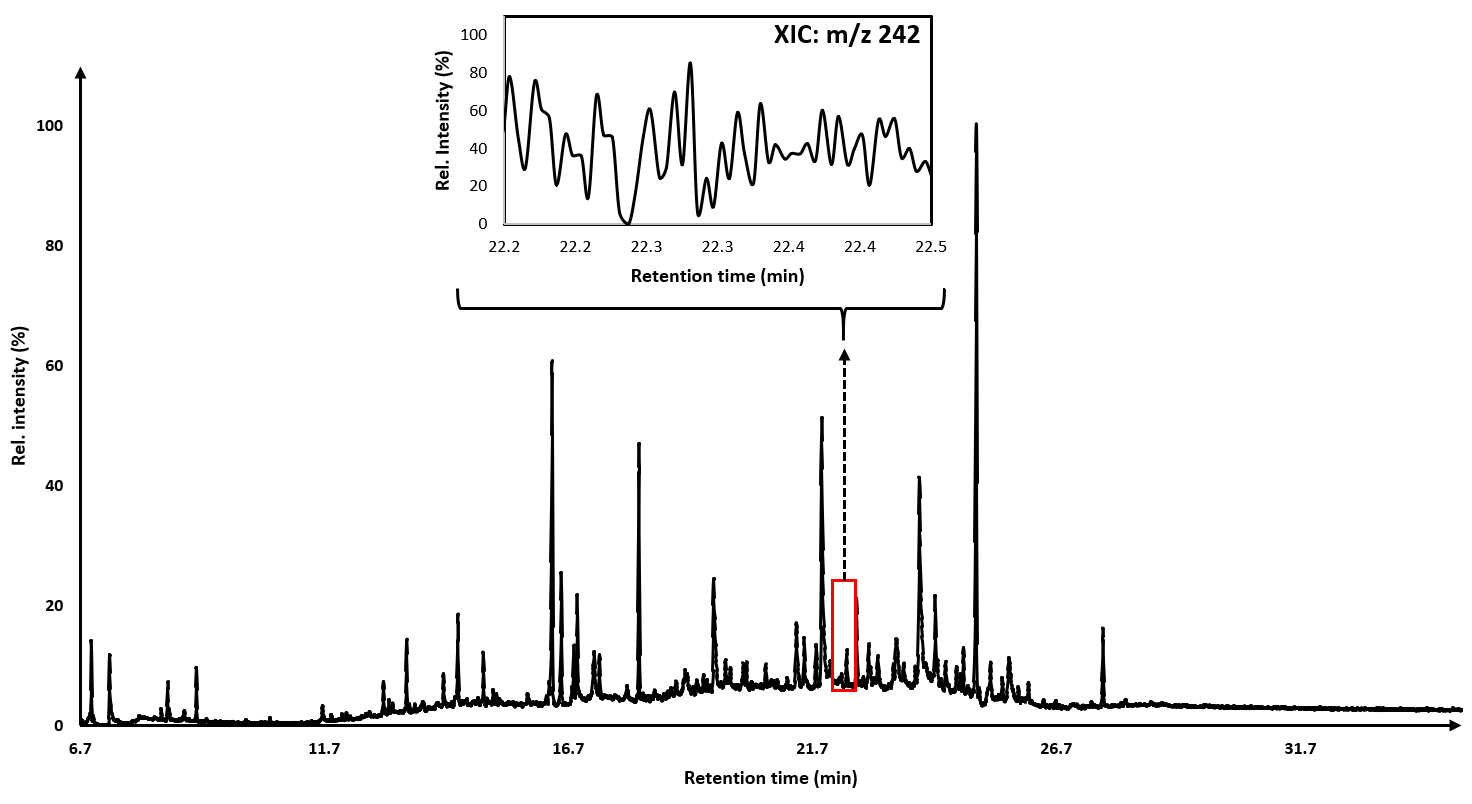


4-nitroaniline

**ATD 175 °C**

**DO3**

**Figure SI-5:** Thermal desorption of Textile 1 at 175°C. The insert shows an extracted ion chromatogram of m/z 242. No peak belonging to Disperse Orange 3 was observed at this desorption temperature.

**Table SI-1:** List of tentatively identified Disperse dyes using reversed-phase liquid chromatography with high-resolution mass spectrometry. The data is processed using an in-house non-target analysis workflow with the commercial software Compound Discoverer 3.0 (Thermo Fisher Scientific, MA, USA). Further details of the analysis can be found in reference 12.

| **Name** | **Formula** | **Calc. MW** | **RT [min]** | **Reference Ion** |
| --- | --- | --- | --- | --- |
| Disperse Red 167:1 | C22 H24 Cl N5 O7 | 505.1356 | 12.1 | [M+H]^+^ |
| Disperse Blue 79:1 (Cl) | C23 H25 Cl N6 O10 | 580.1306 | 11.9 | [M+H]^+^ |
| Disperse Blue 291 | C19 H21 Br N6 O6 | 508.0701 | 13.5 | [M+H]^+^ |
| Disperse Red 179 | C19 H18 N6 O2 S | 394.1203 | 12.1 | [M+H]^+^ |
| Disperse Red 74 | C22 H25 N5 O7 | 471.1743 | 11.1 | [M+H]^+^ |
| Disperse Blue 291 (Cl) | C19 H21 Cl N6 O6 | 464.1205 | 13.4 | [M+H]^+^ |
| Disperse Blue 79:1 | C23 H25 Br N6 O10 | 624.0819 | 12.0 | [M+H]^+^ |
| Disperse Red 153 | C18 H15 Cl2 N5 S | 403.0418 | 13.5 | [M+H]^+^ |
| Disperse Violet 93:1 | C18 H19 Br N6 O5 | 478.0594 | 13.0 | [M+H]^+^ |
| Disperse Blue 291:1 (Cl) | C21 H21 Cl N6 O6 | 488.1206 | 13.6 | [M+H]^+^ |
| Disperse Red 311 | C22 H24 N6 O9 | 516.1598 | 11.2 | [M+H]^+^ |
| Disperse Orange 30 | C19 H17 Cl2 N5 O4 | 449.0649 | 11.8 | [M+H]^+^ |
| Disperse Red 54 | C19 H18 Cl N5 O4 | 415.1041 | 11.7 | [M+H]^+^ |
| Disperse Blue 373 | C21 H21 Br N6 O6 | 532.0705 | 13.7 | [M+H]^+^ |
| Disperse orange 25 | C17 H17 N5 O2 | 323.1377 | 11.7 | [M+H]^+^ |
| Disperse Yellow 211 | C15 H12 Cl N5 O4 | 361.0574 | 7.4 | [M+H]^+^ |
| Disperse Brown 22 | C16 H16 Cl2 N4 O4 | 398.0543 | 10.0 | [M+H]^+^ |
| Disperse Orange 73 | C24 H21 N5 O4 | 443.1589 | 12.4 | [M+H]^+^ |
| Disperse Red 167 | C23 H26 Cl N5 O7 | 519.1516 | 12.6 | [M+H]^+^ |
| Disperse Yellow 119 | C15 H13 N5 O4 | 327.0968 | 10.7 | [M-H] ^-^ |
| Disperse Yellow 126 | C21 H24 N4 O6 | 428.1694 | 10.4 | [M-H] ^-^ |
| Disperse Brown 19 | C20 H20 Br Cl N4 O6 | 526.0276 | 11.5 | [M-H] ^-^ |
| Disperse Violet 33 | C18 H19 N5 O4 | 369.1437 | 11.7 | [M-H] ^-^ |
| Disperse Orange 3 | C12 H10 N4 O2 | 242.0806 | 10.3 | [M-H] ^-^ |
| Disperse Yellow 241 | C14 H10 Cl2 N4 O2 | 336.0184 | 11.5 | [M-H]^-^ |


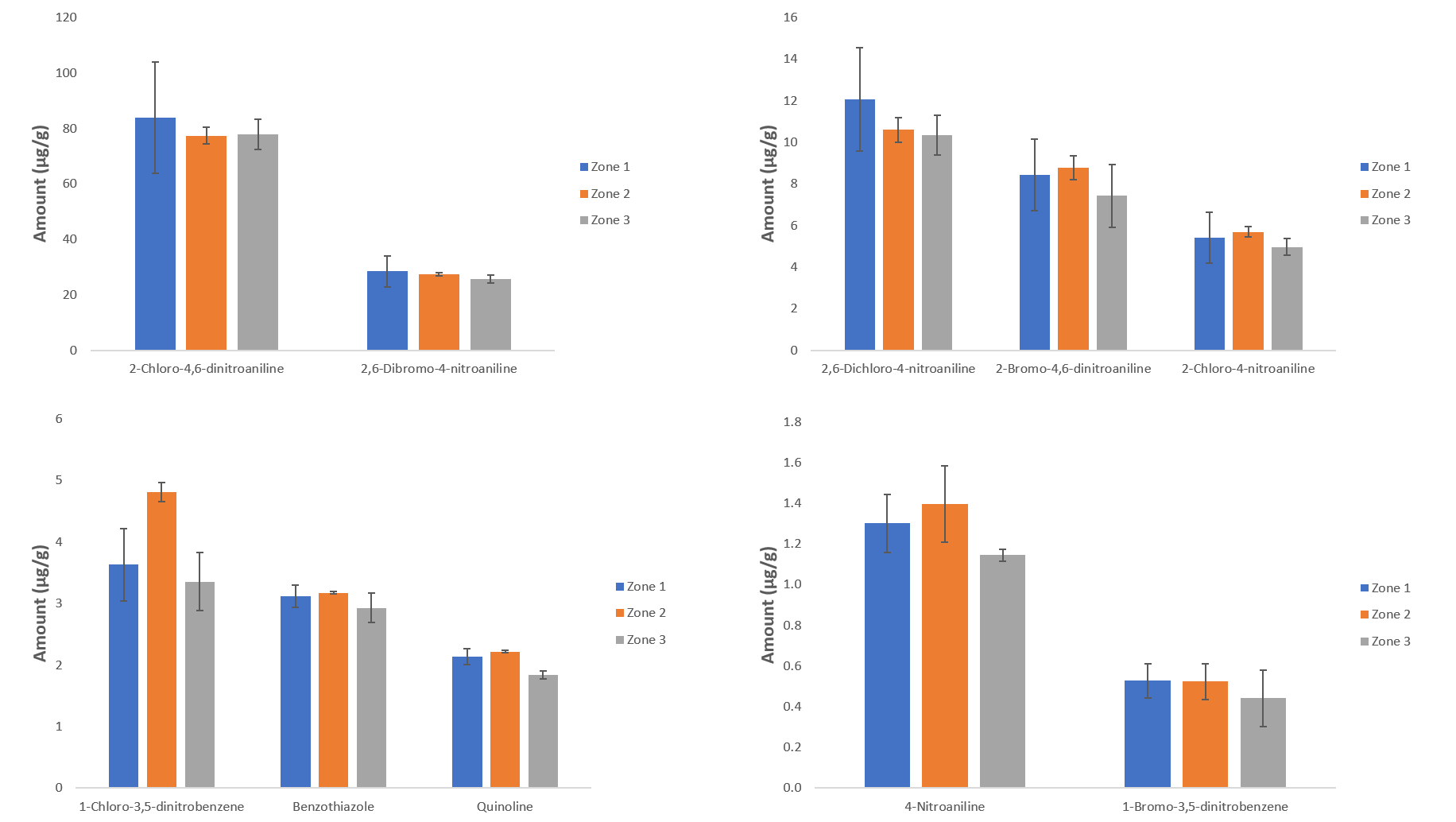


**Figure SI-6:** Thermal desorption using different zones of Textile 1 (N=3).
